# Evaluation of ten composite inflammatory indicators for sarcopenia in type 2 diabetes mellitus

**DOI:** 10.3389/fendo.2026.1926298

**Published:** 2026-08-28

**Authors:** Wen Wei, Luojing Zhong, Hui Cao, Fentao Zhong, Hongze Lin, Hangju Chen, Lingying Cao, Ruiyu Lin, Shidi Wen

**Affiliations:** 1 Department of Endocrinology, Longyan First Affiliated Hospital of Fujian Medical University, Longyan, China; 2 The School of Clinical Medicine, Fujian Medical University, Fuzhou, China; 3 Department of Endocrinology and Rheumatology, Jinjiang Hospital, Jinjiang, China

**Keywords:** composite inflammatory indicators, inflammation, sarcopenia, severe sarcopenia, type 2 diabetes mellitus

## Abstract

**Objective:**

The value of composite inflammatory indicators in identifying sarcopenia in patients with type 2 diabetes mellitus (T2DM) remains unclear. This study investigated associations between ten composite inflammatory indicators and sarcopenia, aiming to identify optimal indicators for sarcopenia detection in T2DM.

**Methods:**

The ten indicators were: systemic immune-inflammation index (SII), systemic inflammation response index (SIRI), neutrophil-to-lymphocyte ratio (NLR), pan-immune inflammation value (PIV), platelet-to-lymphocyte ratio (PLR), advanced lung cancer inflammation index (ALI), neutrophil-to-albumin percentage ratio (NPAR), platelet-to-neutrophil ratio (PNR), neutrophil-lymphocyte-platelet ratio (NLPR), and hemoglobin-to-red cell distribution width ratio (HRR). Associations with sarcopenia and severe sarcopenia were assessed using restricted cubic splines (RCS) and multivariable logistic regression. Receiver operating characteristic (ROC) curves evaluated their identification ability.

**Results:**

Sarcopenia prevalence was 34.1% (*n* = 261); severe sarcopenia was 11.5% (*n* = 78). RCS analysis showed positive correlations of SII, SIRI, NLR, PIV, PLR, NPAR, and NLPR with sarcopenia, while ALI, PNR, and HRR showed inverse trends. Multivariable regression confirmed positive associations for SII, SIRI, NLR, PIV, NPAR, NLPR, and an inverse association for ALI (all *P* < 0.05). The area under the ROC curve (AUC) for ALI in identifying sarcopenia and severe sarcopenia was 0.68 and 0.70, respectively.

**Conclusion:**

Elevated SII, SIRI, PIV, NPAR, NLPR, and NLR were positively associated with sarcopenia in T2DM, while ALI was inversely associated. Among all composite inflammatory indicators, ALI demonstrated the best ability to identify sarcopenia and severe sarcopenia in T2DM patients.

## 1 Introduction

Sarcopenia is characterized by the progressive loss of skeletal muscle mass and function, resulting in impaired physical performance and mobility (1). This age-related condition is linked to increased risks of adverse outcomes (e.g., falls, frailty, disability and osteoporosis), diminished quality of life, and elevated mortality (2, 3). Evidence confirms type 2 diabetes mellitus (T2DM) as an independent risk factor for sarcopenia (4). The prevalence of sarcopenia in T2DM patients is 18%, which is substantially higher than that in the non-diabetic population (4). Compared with T2DM patients without sarcopenia, those with sarcopenia had higher risks of chronic kidney disease (CKD), diabetic retinopathy (DR), diabetic peripheral neuropathy (DPN), atherosclerosis, cognitive impairment, infection, and mortality (5–8). Thus, early identification and management of sarcopenia are critical for improving clinical outcomes and quality of life in T2DM patients.

Multiple composite inflammatory indicators, such as the systemic immune-inflammation index (SII), systemic inflammation response index (SIRI), neutrophil-to-lymphocyte ratio (NLR), pan-immune inflammation value (PIV), platelet-to-lymphocyte ratio (PLR), and advanced lung cancer inflammation index (ALI) have demonstrated significant predictive value in identifying the risk of various chronic inflammatory diseases (9–16). Additionally, these inflammatory indicators such as SII, SIRI, NLR, PIV, PLR, and ALI have been shown to be significantly associated with sarcopenia in the general population, as well as in patients with hypertension, oncology cohorts, and older adults (9–12). Studies have shown that the systemic inflammatory index neutrophil-lymphocyte-platelet ratio (NLPR) serves as a reliable inflammatory biomarker for predicting the development of metabolic syndrome in patients with T2DM (17). In a prospective cohort study, NLPR was found to be an independent predictor of adverse outcomes in patients with acute coronary syndrome, and elevated NLPR levels were associated with a higher rate of major adverse cardiovascular events (18). A retrospective case-control study showed that NLPR outperformed indicators such as SII, SIRI, and NLR in assessing atherosclerotic risk (19). Meanwhile, the neutrophil percentage-to-albumin ratio (NPAR), which integrates albumin levels with hematological parameters, demonstrated superior predictive power for heart failure and diabetic microvascular complications compared to NLR (20–22). The composite inflammatory index platelet-to-neutrophil ratio (PNR) have been shown to predict mortality in patients with ischemic stroke (23). A retrospective analysis showed that low PNR was significantly associated with lower extremity artery disease in T2DM patients (24). Song et al. found that PNR exhibited better prognostic performance than SII, PLR, and NLR in ovarian cancer patients (25). Nevertheless, there is currently a lack of studies investigating the association between these inflammatory indicators and sarcopenia in patients with T2DM. Given the established performance of NLPR, NPAR and PNR in other chronic inflammatory diseases, we hypothesize that these composite markers may also hold potential as identifying tools for sarcopenia in patients with T2DM.

Red cell distribution width (RDW) and hemoglobin (Hb) are important parameters in routine clinical blood testing. The hemoglobin-to-red cell distribution width ratio (HRR) is a composite indicator that integrates Hb and RDW, enabling a comprehensive assessment of inflammatory status, nutritional status, and oxygen metabolism in chronic diseases (26). Previous studies have shown that HRR is associated with poor prognosis across a range of conditions, including cardiovascular disease, chronic kidney disease, cancer, and depression (27–30). A retrospective cross-sectional analysis revealed that HRR may serve as a potential auxiliary screening tool for the early identification of populations at risk of sarcopenia, and that lower HRR levels were significantly associated with an increased risk of sarcopenia (31). However, studies on the association between HRR and sarcopenia in T2DM patients are still limited.

Therefore, the aim of this study is to elucidate the relationship between composite inflammatory indicators (SII, SIRI, NLR, PIV, NLPR, NPAR, PLR, ALI, PNR, HRR) and sarcopenia in T2DM, so as to select the best inflammatory indicators and provide a basis for early identification of sarcopenia and risk stratification in patients with type 2 diabetes.

## 2 Methods

### 2.1 Study population

**Figure 1:**
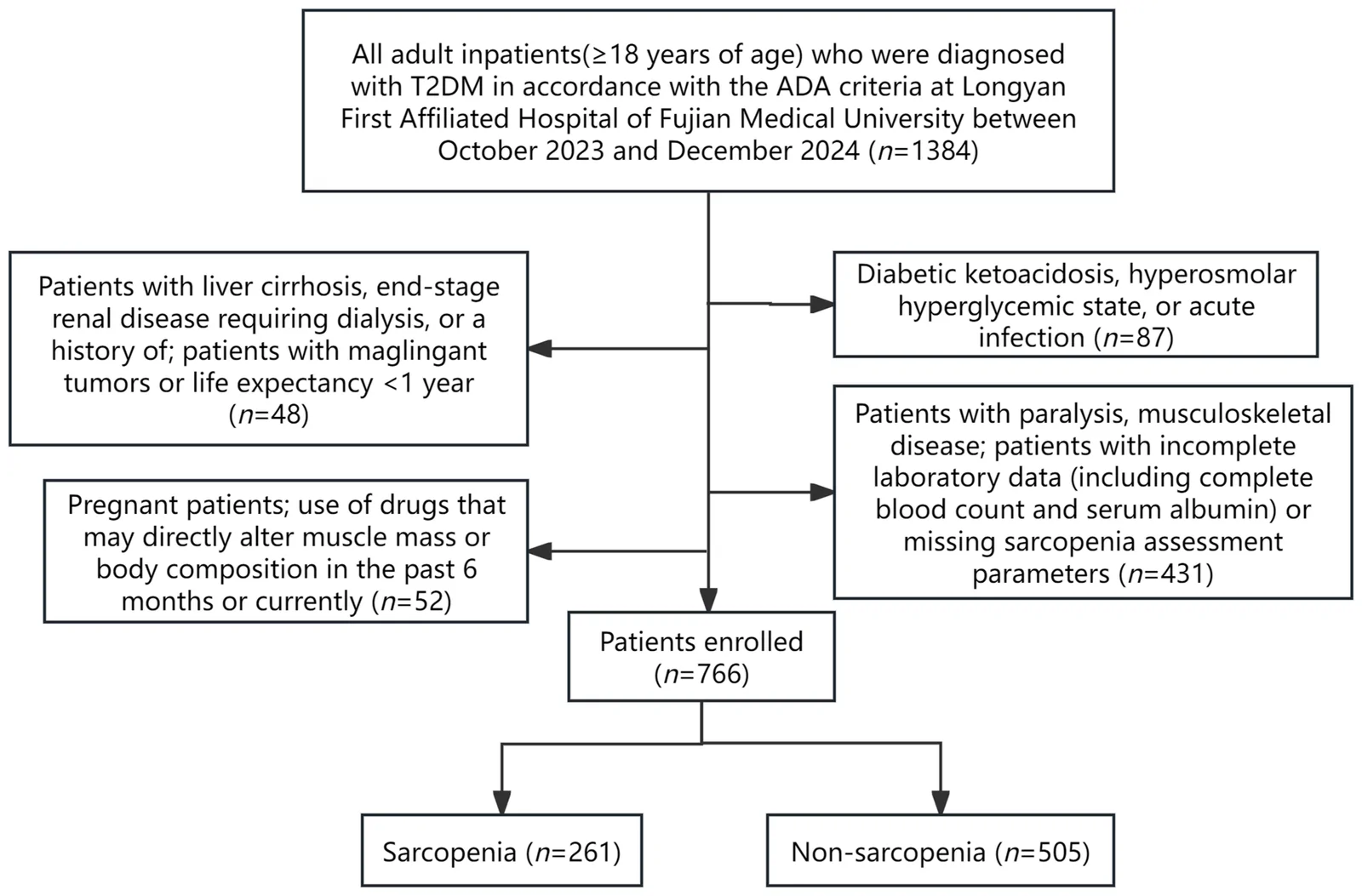
The flow of participants through the trial.

This cross-sectional study included adult inpatients aged ≥ 18 years with T2DM, who were admitted to Longyan First Affiliated Hospital of Fujian Medical University, Fujian, China, between October 2023 and December 2024. T2DM was diagnosed according to the American Diabetes Association (ADA) criteria. The exclusion criteria included (1): patients presenting with acute diabetes-related complications, including diabetic ketoacidosis, hyperosmolar hyperglycemic state, or acute infection (2); patients with liver cirrhosis, end-stage renal disease requiring dialysis, or a history of (3); pregnant patients (4); patients with malignant tumors or life expectancy <1 year (5); use of drugs (e.g, corticosteroids, diuretics) that may directly alter muscle mass or body composition in the past 6 months or currently (6); patients with paralysis, musculoskeletal disease (7); patients with incomplete laboratory data (including complete blood count and serum albumin) or missing sarcopenia assessment parameters. Written informed consent was obtained from all participants. Of the 1384 patients initially screened, 766 met the eligibility criteria and were included in the final analysis (**Figure 1**).

### 2.2 Data collection and clinical definitions

Trained personnel collected demographic characteristics and health-related data using standardized questionnaires, covering age, sex, smoking status, alcohol consumption, and medical history. Self-reported health conditions encompassed physician-diagnosed hypertension (HT), cardiovascular disease (CVD), and stroke. Concurrently, upon hospital admission, standardized measurements of height, weight, and blood pressure (BP) were conducted by the nurse. Furthermore, a comprehensive review of patient data from the electronic medical record system was conducted. Venous blood samples were collected in the morning following an 8-hour overnight fast.

Low education was defined as a highest educational attainment of high school diploma or lower. According to the Guideline for the management of diabetes mellitus in the elderly in China (2024 edition), moderate-intensity exercise was defined as: 40%-59% of heart rate reserve, a perceived exertion of being somewhat strenuous, and accelerated but not rapid heartbeat and respiration (32, 33). High-intensity exercise was defined as activity exceeding these criteria. Accordingly, we categorized physical activity into three groups: no exercise, low-intensity (below the moderate-intensity threshold), and moderate-to-high-intensity (meeting or exceeding the moderate- intensity threshold, combining both moderate and high-intensity exercise). The smoking status was categorized as either “no current smoking” or “current smoking”. The classification of drinking was based on the categories of “no current drinking” or “current drinking”. Body mass index (BMI, kg/m²) was calculated as weight (kg) divided by height squared (m²). DR was identified using ophthalmological examination findings and defined according to the International Clinical Diabetic Retinopathy Disease Severity Scale (34). The diagnosis of diabetic nephropathy (DN) followed the Kidney Disease Improving Global Outcomes (KDIGO) guidelines (35). DPN was defined in accordance with the Chinese clinical practice guidelines for the management of type 2 diabetes (36). CVD encompassed stroke and/or coronary artery disease (CAD). Osteoporosis was defined as a bone density at the hip or lumbar spine that was 2.5 standard deviations or more below the mean bone mineral density of a reference population (T-score ≤ -2.5) (37).

### 2.3 Assessment of composite inflammatory indicators

Complete blood count (CBC) count, including white blood cell (WBC) count, neutrophil count, lymphocyte count, and monocyte count (expressed in 10^^9^ cells/L), were assessed using flow cytometry. Hemoglobin concentration and RDW were measured using the Beckman Coulter DxH 900 automated hematology analyzer. Serum albumin concentrations were measured using the bromocresol green method. Through blood routine test, serum albumin, and BMI to calculate these following composite inflammatory indicators: SII, SIRI, NLR, PIV, PLR, ALI, NPAR, PNR, NLPR, and HRR using the following formulas (9, 19, 25, 31, 38–41):


SII=platelet count × neutrophil count(10^9cells/L)/lymphocyte count(10^9cells/L),

SIRI=neutrophil count × monocyte count(10^9cells/L)/lymphocyte count(10^9cells/L),

NLR=neutrophil count/lymphocyte count(10^9cells/L),

$$\text{PIV}=\text{neutrophil count}(10^{9}\text{cells/L})\times \text{ platelet count}(10^{9}\text{cells/L})\times \text{ monocyte count}(10^{9}\text{cells/L})/\text{lymphocyte count}(10^{9}\text{cells/L}),$$

PLR=platelet count(10^9cells/L)/lymphocyte count(10^9cells/L)

NPAR=neutrophil percentage (%)×100/albumin (g/dL),

NLPR=neutrophil count(10^9cells/L)× 100/lymphocyte count(10^9cells/L)/platelet count(10^9cells/L),

PNR=platelet count(10^9cells/L)/neutrophil count(10^9cells/L),

HRR=hemoglobin (g/L)/RDW(%),

$$\text{ALI}=\left(\text{BMI, kg/m}{\text{BMI, kg/m}}^{2}\right)\times \text{ albumin (g/dL)}/\text{NLR}$$

### 2.4 Assessment of sarcopenia

Body composition was assessed by trained technicians in the Department of Nuclear Medicine using dual-energy X-ray absorptiometry (DXA; Hologic Discovery, Waltham, MA, USA). Skeletal muscle mass was evaluated via DXA. Muscle strength was measured using a hydraulic hand dynamometer (EH101; CAMRY, Guangdong, China). Participants were seated with their elbows flexed at 90°, and two measurements were taken on the dominant hand, with the maximum value recorded. The 6-meter walking speed was assessed as follows: participants walked at their habitual pace over a 6-meter course. Timing began once the participant initiated movement, and the time to complete the distance without deceleration was recorded. The final walking speed was calculated as the average of at least two trials.

Skeletal muscle mass index (SMI, kg/m²) was calculated as appendicular skeletal muscle mass (ASM, kg) divided by height squared (m²). Based on the Asian Working Group for Sarcopenia (AWGS) consensus guidelines, low muscle mass was defined as an SMI < 5.4 kg/m² for women and < 7.0 kg/m² for men. Low muscle strength was defined as handgrip strength < 18.0 kg for women and < 28.0 kg for men. Low physical performance was defined as a 6-meter walking speed < 1.0 m/s. Sarcopenia was defined according to the AWGS 2019 guidelines as the presence of low muscle mass, low muscle strength, or poor physical performance (42). Severe sarcopenia was defined as having both low muscle mass and low muscle strength, as well as poor physical performance (43).

### 2.5 Statistical analysis

Continuous variables were presented as means ± standard deviation or as median and interquartile range based on the distribution of the data. Categorical variables were presented as number (%). For group comparisons, continuous variables were analyzed using the Student’s t-test or Wilcoxon rank-sum test, as appropriate. Categorical variables were compared using the chi-square test.

We compared the prevalence of T2DM patients with sarcopenia and severe sarcopenia according to tertiles of composite inflammatory indicators using chi-square tests. Restricted cubic splines (RCS) were used to detect the association between composite inflammatory indicators and both sarcopenia and severe sarcopenia. We positioned three nodes at the 10th, 50th, and 90th percentiles of ten composite inflammatory indicators to construct RCS analysis. The median values of ten composite inflammatory indicators were defined as the reference, with an odds ratio (OR) of 1.0. The variables included in the spline models were identical to the independent variables examined in the univariate logistic regression analysis.

Univariable and multivariable logistic regression models were employed to examine the associations between composite inflammatory indicators and both sarcopenia and severe sarcopenia. The results of the logistic regression analysis were presented as ORs and 95% confidence intervals (CIs). To account for multiple comparisons across the two multivariable logistic regression models (10 inflammatory indicators per model, totaling 20 tests), we applied Bonferroni correction, with the statistical significance threshold set at *P* < 0.0025 (0.05/20). Only associations with *P* < 0.0025 were considered statistically significant. For sarcopenia, the full model was adjusted for: age≥60, sex, low education, exercise, BMI≥25, smoking, drinking, duration of diabetes, HT, DR, DN, DPN, CVD, low-density lipoprotein cholesterol (LDL-C), high-density lipoprotein cholesterol (HDL-C), osteoporosis and sodium-glucose- linked transporter-2 inhibitors (SGLT-2i)/glucagon-like peptide-1 receptor agonists (GLP-1RA). For severe sarcopenia, the full model was adjusted for: age≥60, sex, low education, BMI≥25, duration of diabetes, CVD and osteoporosis.

The performance of composite inflammatory indicators in identifying sarcopenia and severe sarcopenia in patients with T2DM was assessed using receiver operating characteristic (ROC) curves analysis. Pairwise comparisons of ROC curves were performed using DeLong’s test to compare the area under the curve (AUC) of ALI with each of the other nine composite inflammatory indicators. To account for multiple comparisons across two outcomes and nine pairwise comparisons per outcome (18 tests in total), Bonferroni correction was applied, with statistical significance set at *P* < 0.0028 (0.05/18). Bootstrap internal validation was performed using 1,000 resamples with replacement to assess the stability of the optimal ALI cutoffs. The 95% confidence intervals for the cutoffs were derived from the 2.5th and 97.5th percentiles of the bootstrap distribution. Decision curve analysis (DCA) was performed to evaluate the clinical net benefit of the ALI for predicting sarcopenia and severe sarcopenia. Net benefit was calculated across threshold probabilities ranging from 0 to 1, and the ALI-included model was compared with the “treat-all” and “treat-none” strategies.

All statistical analyses were performed using R,version 4.0.3 software (R Foundation for Statistical Computing, Vienna, Austria) and SPSS 25.0 software (IBM Corporation, Armonk, NY, USA), with statistical significance defined as *P* value < 0.05.

## 3 Results

### 3.1 Baseline characteristics of the participants

The characteristics of the participants according to the presence of sarcopenia were shown in **Table 1**. The study included 766 patients diagnosed with T2DM, with an average age of 58.1 years, and 57.7% were males. The median duration of diabetes was 6.0 years and the glycosylated hemoglobin (HbA1c) was 9.8% ± 2.3. Almost half of the patients had HT (46.1%; *n* = 353), 25.1% (*n* = 183) had DR, 11.0% (*n* = 84) had DN, 35.3% (*n* = 270) had DPN, 4.6% (*n* = 35) had stroke, 10.4% (*n* = 80) had CVD and 30.7% (*n* = 235) had osteoporosis.

**Table 1:** Baseline characteristics of the participants.

Characteristic	Total (*n* = 766)	Non-sarcopenia (*n* = 505)	Sarcopenia (*n* = 261)	*P*-value
Demographic characteristics				
Age (years)	58.1 ± 12.6	54.5 ± 11.5	65.2 ± 11.6	<0.001
Age ≥ 60, *n* (%)	336 (43.9)	157 (31.1)	179 (68.6)	<0.001
Male, *n* (%)	442 (57.7)	288 (57.0)	154 (59.0)	0.655
Low education, *n* (%)	647 (84.8)	415 (82.5)	232 (89.2)	0.019
Exercise, *n* (%)				0.051
No, *n* (%)	145 (20.5)	99 (22.1)	46 (17.8)	
Low intensity, *n* (%)	361 (51.1)	213 (47.7)	148 (57.1)	
Moderate to high intensity, *n* (%)	200 (28.3)	135 (30.2)	65 (25.1)	
BMI (kg/m^2^)	24.56 ± 3.63	25.48 ± 3.64	22.79 ± 2.88	<0.001
BMI ≥ 25 kg/m^2^, *n* (%)	320 (41.8)	264 (52.3)	56 (21.5)	<0.001
Smoking, *n* (%)	186 (24.3)	133 (26.3)	53 (20.3)	0.079
Drinking, *n* (%)	350 (45.7)	256 (50.7)	94 (36.0)	<0.001
Medical history and clinical condition				
Hypertension, *n* (%)	353 (46.1)	219 (43.4)	134 (51.3)	0.043
SBP (mmHg)	133.8 ± 18.4	133.2 ± 18.0	134.9 ± 19.1	0.247
DBP (mmHg)	82.9 ± 11.8	83.9 ± 11.9	81.1 ± 11.6	0.002
Duration of diabetes (years)	6.0 (0.5, 10.0)	5.0 (0.1, 10.0)	8.0 (2.0,13.0)	<0.001
DR, *n* (%)	183 (25.1)	108 (22.0)	75 (31.6)	0.007
DN, *n* (%)	84 (11.0)	47 (9.3)	37 (14.2)	0.052
DPN, *n* (%)	270 (35.3)	163 (32.3)	107 (41.2)	0.019
Stroke, *n* (%)	35 (4.6)	16 (3.2)	19 (7.3)	0.016
CAD, *n* (%)	49 (6.4)	27 (5.3)	22 (8.4)	0.134
CVD, *n* (%)	80 (10.4)	43 (8.5)	37 (14.2)	0.021
Osteoporosis, *n* (%)	235 (30.7)	125 (24.8)	110 (42.1)	<0.001
Laboratory examination				
FBG (mmol/L)	8.64 ± 3.24	8.72 ± 3.27	8.47 ± 3.18	0.312
HbA1C (%)	9.77 ± 2.29	9.67 ± 2.22	9.95 ± 2.42	0.113
Albumin (g/L)	40.63 ± 4.21	41.36 ± 3.91	39.22 ± 4.42	<0.001
TC (mmol/L)	5.18 ± 1.47	5.27 ± 1.43	5.02 ± 1.51	0.027
TG (mmol/L)	1.63 (1.11, 2.60)	1.77 (1.18, 2.83)	1.49 (0.98,2.14)	<0.001
HDL-C (mmol/L)	1.12 ± 0.28	1.10 ± 0.27	1.15 ± 0.29	0.027
LDL-C (mmol/L)	3.25 ± 1.01	3.30 ± 0.97	3.15 ± 1.07	0.043
Creatinine (umol/L)	68.0 (56.0,84.0)	67.0 (55.0,82.0)	70.0 (58.0,91.0)	<0.048
Vitamin D (nmol/L)	59.82 ± 18.68	59.06 ± 18.36	61.30 ± 19.24	0.128
WBC (10^9^/L)	6.99 ± 2.07	6.83 ± 1.77	7.31 ± 2.53	0.003
NEU (10^9^/L)	4.31 ± 1.83	4.09 ± 1.40	4.73 ± 2.40	<0.001
LYM (10^9^/L)	2.06 ± 0.70	2.13 ± 0.69	1.93 ± 0.70	<0.001
MON (10^9^/L)	0.43 ± 0.16	0.42 ± 0.15	0.45 ± 0.17	0.008
Platelet (10^9^/L)	229.94± 63.06	230.56 ± 60.66	228.75 ± 67.56	0.707
Hb (g/L)	139.39 ± 17.89	142.28 ± 17.43	133.81 ± 17.48	<0.001
RDW (%)	12.82 ± 1.33	12.81 ± 1.39	12.83 ± 1.21	0.840
Inflammatory indicators				
SII	437.90 (312.18, 647.15)	416.11 (306.09, 582.31)	514.59 (329.97, 774.51)	<0.001
SIRI	0.79 (0.54,1.22)	0.75 (0.51,1.07)	0.93 (0.60,1.54)	<0.001
NLR	1.98 (1.48,2.69)	1.86 (1.46,2.48)	2.23 (1.57,3.42)	<0.001
PIV	172.49 (114.16, 286.44)	161.55 (108.86, 255.37)	207.09 (124.98, 374.03))	<0.001
PLR	112.32 (88.18, 143.82)	107.41 (85.76, 137.32)	118.93 (94.74, 155.84)	<0.001
NPAR	14.61 (12.93, 16.78)	14.25 (12.57, 16.08)	15.71 (13.61, 18.05)	<0.001
NLPR	0.89 (0.65,1.31)	0.83 (0.62,1.16)	1.04 (0.69,1.56)	<0.001
PNR	55.94 (43.27, 70.76)	57.83 (45.71, 73.03)	51.54 (39.48, 66.91)	<0.001
HRR	11.00 ± 1.86	11.24 ± 1.85	10.55 ± 1.79	<0.001
ALI	51.45 (34.68, 69.19)	55.90 (41.01, 75.07)	40.68 (26.69, 57.87)	<0.001
Medication				
SGLT-2i	79 (10.6)	52 (10.6)	27 (10.8)	0.998
GLP-1RA	32 (4.3)	22 (4.5)	10 (4.0)	0.901
SGLT-2i/GLP-1RA	101 (13.6)	67 (13.6)	34 (13.5)	0.996
Insulin	129 (17.4)	77 (15.7)	52 (20.8)	0.102
Statins	89 (12.8)	62 (13.3)	27 (11.7)	0.637

BMI, body mass index; SBP, systolic blood pressure; DBP, diastolic blood pressure; DR, diabetic retinopathy; DN, diabetic nephropathy; DPN, diabetic peripheral neuropathy; CAD, coronary artery disease; CVD, Cardiovascular disease; FBG, fasting blood glucose; HbA1c, glycosylated hemoglobin; TC, total cholesterol; TG, triglycerides; HDL-C, high-density lipoprotein cholesterol; LDL-C, low-density lipoprotein cholesterol; WBC, white blood cell count; NEU, neutrophil count; LYM, lymphocyte count; MON, monocyte count; Hb, hemoglobin; RDW, red blood cell distribution width; SII, systemic immune- inflammation index; SIRI, systemic inflammation response index; NLR, neutrophil-to-lymphocyte ratio; PIV, pan-immune inflammation value; PLR, platelet-to-lymphocyte ratio; NPAR, neutrophil-to-albumin percentage ratio; NLPR, neutrophil-lymphocyte-platelet ratio; PNR, platelet-to-neutrophil ratio; HRR, hemoglobin- to-red cell distribution width ratio; ALI, advanced lung cancer inflammation index; SGLT-2i, sodium-glucose-linked transporter-2 inhibitor; GLP-1RA, glucagon-like peptide-1 receptor agonists; SGLT-2i/GLP-1RA, use of either sodium-glucose-linked transporter-2 inhibitors or glucagon-like peptide-1 receptor agonists.

As shown in **Table 1**, 261 patients (34.1%) met the criteria for sarcopenia, and 78 patients (11.5%) met the criteria for severe sarcopenia. Compared with patients without sarcopenia, patients with sarcopenia were more likely to be older, possess lower educational, and lower BMI (*P* < 0.05). Additionally, sarcopenic patients had a higher prevalence of DR, DPN, stroke, CVD, and osteoporosis (*P* < 0.05). Patients with sarcopenia exhibited elevated levels of WBC, neutrophils, monocytes, lymphocytes and composite inflammation indicators (SII, SIRI, NLR, PIV, PLR, NPAR, and NLPR) than patients without sarcopenia, alongside reduced ALI, PNR and HRR (*P* < 0.01).

**Figure 2:**
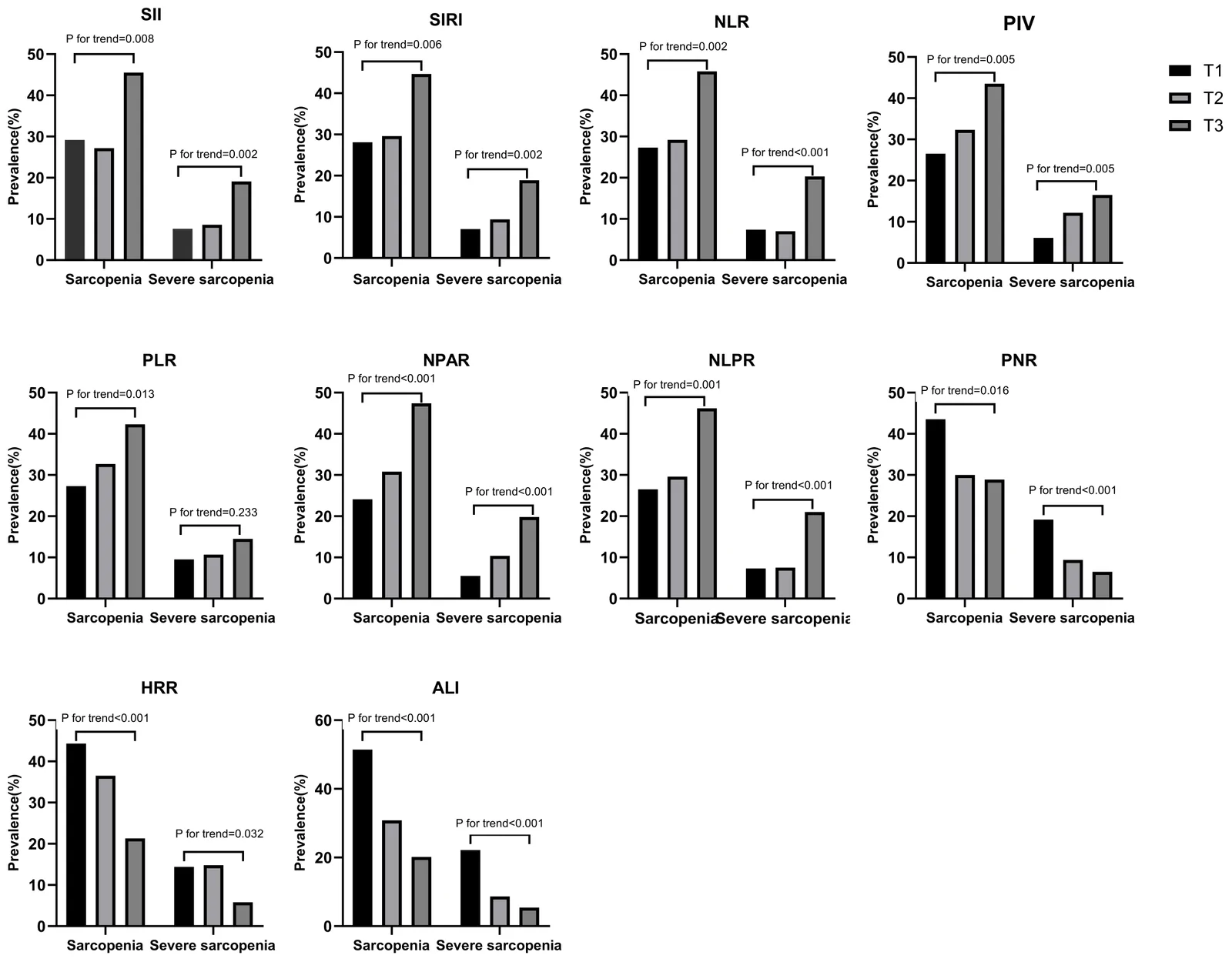
Prevalence of T2DM patients with sarcopenia and severe sarcopenia across the composite inflammatory indicators tertiles. T1, the 1^st^ tertiles, T2, the 2^nd^ tertiles, T3, the 3^rd^ tertiles, SII, systemic immune-inflammation index, SIRI, systemic inflammation response index; NLR, neutrophil-to-lymphocyte ratio; PIV, pan-immune inflammation value; PLR, platelet- to-lymphocyte ratio; NPAR, neutrophil-to-albumin percentage ratio; NLPR, neutrophil-lymphocyte-platelet ratio; PNR, platelet-to-neutrophil ratio; HRR, hemoglobin-to-red cell distribution width ratio; ALI, advanced lung cancer inflammation index; T2DM, type 2 diabetes mellitus.

The prevalence of sarcopenia and severe sarcopenia significantly increased from the lowest to highest tertiles of SII, SIRI, NLR, PIV, NPAR, and NLPR (*P* for trend < 0.01). Conversely, the prevalence of sarcopenia and severe sarcopenia significantly decreased from the lowest to highest tertiles of ALI, PNR and HRR (*P* for trend < 0.05). However, for PLR, the prevalence of sarcopenia significantly increased from the lowest to highest tertiles (*P* for trend < 0.05), while the prevalence of severe sarcopenia exhibited increasing trend from the lowest to highest tertiles (*P* for trend = 0.233) (**Figure 2**).

### 3.2 RCS analysis investigated the relationship between inflammatory indicators levels and both sarcopenia and severe sarcopenia

RCS analysis showed positive linear correlations between SII, SIRI, NLR, PIV, PLR, NPAR, NLPR and the odds of sarcopenia (all *P* for nonlinearity > 0.05). However, the levels of ALI, PNR and HRR showed an inverse trend with the odds of sarcopenia (all *P* for nonlinearity < 0.05).

**Figure 3:**
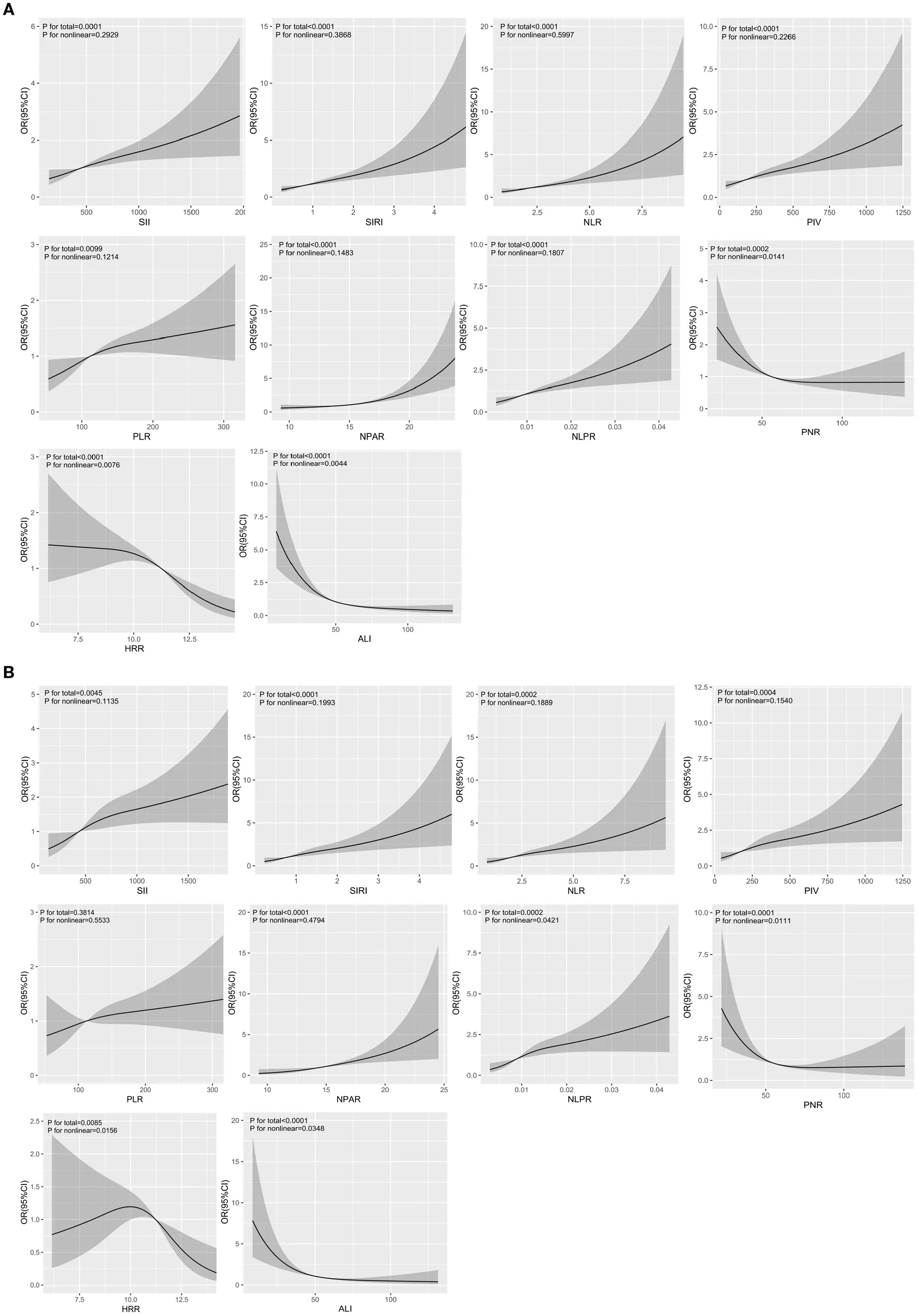
Restricted cubic splines of the composite inflammatory indicators odds ratio of sarcopenia and severe sarcopenia in patients with T2DM. **(A)** The restricted cubic spline of the composite inflammatory indicators odds ratio of sarcopenia. **(B)** The restricted cubic spline of the composite inflammatory indicators odds ratio of severe sarcopenia. SII, systemic immune-inflammation index; SIRI, systemic inflammation response index; NLR, neutrophil-to-lymphocyte ratio; PIV, pan- immune inflammation value; PLR, platelet-to-lymphocyte ratio; NPAR, neutrophil- to-albumin percentage ratio; NLPR, neutrophil-lymphocyte-platelet ratio; PNR, platelet-to-neutrophil ratio; HRR, hemoglobin-to-red cell distribution width ratio; ALI, advanced lung cancer inflammation index; T2DM, type 2 diabetes mellitus.

Similarly, positive linear correlations were observed between SII, SIRI, NLR, PIV, PLR, NPAR and the odds of severe sarcopenia (all *P* for nonlinearity > 0.05). The level of NLPR showed a positive trend with the odds of severe sarcopenia, while the level of ALI, PNR and HRR showed an inverse trend with the odds of severe sarcopenia (all *P* for nonlinearity < 0.05) (**Figure 3**).

### 3.3 Association of inflammatory indicators with sarcopenia by logistic regression analysis

The univariable analysis showed that SII, SIRI, PIV, NPAR, NLPR, PLR and NLR were positively associated with sarcopenia (*P* < 0.05), while ALI, PNR and HRR were negatively associated with sarcopenia (*P* < 0.01). We further converted inflammatory indicators from continuous variable to categorical variable (tertiles). Compared to the T1 group of SII, SIRI, PIV, NPAR, NLPR, PLR, and NLR, patients in the T3 group had a significantly higher odds of sarcopenia (*P* < 0.01). In contrast, compared to the T1 group of HRR, patients in the T3 group showed significantly lower odds of sarcopenia (*P* < 0.01). Similarly, compared to the T1 groups of ALI and PNR, both the T2 and T3 groups exhibited significantly lower odds of sarcopenia (*P* < 0.01).

Multivariable regression analysis revealed that SII, SIRI, PIV, NPAR, NLPR, and NLR were positively associated with sarcopenia (*P* < 0.05). In contrast, ALI demonstrated a negative association with sarcopenia (*P* < 0.05). After Bonferroni correction (threshold: *P* < 0.0025), ALI remained significantly associated with sarcopenia. When inflammatory indicators were analyzed as categorical variable, after full adjustment, compared to T1 group of SII, SIRI, PIV, PLR, and NLR, patients in the T3 group showed significant correlations with sarcopenia, except for NPAR, NLPR, PNR and HRR (*P* < 0.05). Especially, compared to T1, only ALI in T2 and T3 exhibited significant correlations with sarcopenia (*P* < 0.05) (**Table 2**).

**Table 2:** Association between inflammatory indicators and sarcopenia in T2DM patients.

Variables	Model 1		Model 2		Model 3	
	OR (95%CI)	*P*-value	OR (95%CI)	*P*-value	OR (95%CI)	*P*-value
SII (continuous)	1.01(1.00,1.02)	<0.001	1.01(1.00,1.02)	0.001	1.01(1.00,1.02)	0.012
Tertiles of SII						
T1	Reference		Reference		Reference	
T2	0.93(0.63,1.36)	0.696	1.00(0.67,1.51)	0.990	0.93(0.58,1.50)	0.766
T3	2.02(1.40,2.92)	<0.001	1.84(1.25,2.74)	0.002	2.07(1.29,3.35)	0.003
SIRI (continuous)	1.66(1.39,2.03)	<0.001	1.54(1.27,1.89)	<0.001	1.49(1.20,1.89)	0.001*
Tertiles of SIRI						
T1	Reference		Reference		Reference	
T2	1.08(0.74,1.58)	0.698	0.98(0.65,1.47)	0.912	1.06(0.65,1.73)	0.813
T3	2.07(1.43,3.00)	<0.001	1.58(1.05,2.38)	0.027	1.82(1.12,2.96)	0.016
NLR (continuous)	1.32(1.19,1.49)	<0.001	1.23(1.11,1.38)	<0.001	1.20(1.08,1.36)	0.002*
Tertiles of NLR						
T1	Reference		Reference		Reference	
T2	1.10(0.75,1.62)	0.622	1.02(0.68,1.54)	0.910	1.09(0.68,1.77)	0.716
T3	2.26(1.56,3.28)	<0.001	1.69(1.14,2.54)	0.010	1.65(1.02,2.66)	0.040
PIV (continuous)	1.01(1.00,1.02)	<0.001	1.01(1.00,1.02)	<0.001	1.01(1.00,1.02)	0.002*
Tertiles of PIV						
T1	Reference		Reference		Reference	
T2	1.32(0.91,1.94)	0.148	1.42(0.95,2.14)	0.092	1.45(0.90,2.35)	0.130
T3	2.14(1.47,3.11)	<0.001	2.05(1.37,3.09)	<0.001	2.33(1.44,3.82)	0.001*
PLR (continuous)	1.01(1.00,1.02)	0.013	1.01(1.00,1.02)	0.037	1.01(1.00,1.02)	0.116
Tertiles of PLR						
T1	Reference		Reference		Reference	
T2	1.30(0.89,1.90)	0.181	1.33(0.89,2.00)	0.163	1.30(0.80,2.11)	0.289
T3	1.95(1.35,2.84)	<0.001	1.87(1.26,2.79)	0.002	1.80(1.11,2.93)	0.017
NPAR (continuous)	1.20(1.14,1.27)	<0.001	1.14(1.08,1.20)	<0.001	1.11(1.04,1.18)	0.001*
Tertiles of NPAR						
T1	Reference		Reference		Reference	
T2	1.40(0.95,2.07)	0.092	1.18(0.78,1.78)	0.444	1.09(0.68,1.78)	0.715
T3	2.84(1.95,4.17)	<0.001	1.89(1.26,2.85)	0.002	1.62(1.00,2.63)	0.051
NLPR (continuous)	1.64(1.35,2.05)	<0.001	1.39(1.14,1.73)	0.003	1.36(1.09,1.73)	0.010
Tertiles of NLPR						
T1	Reference		Reference		Reference	
T2	1.17(0.79,1.72)	0.430	0.99(0.66,1.50)	0.976	0.97(0.60,1.57)	0.899
T3	2.39(1.65,3.48)	<0.001	1.50(1.00,2.27)	0.053	1.43(0.89,2.31)	0.143
PNR (continuous)	0.99(0.98,1.00)	0.001	0.99(0.99,1.00)	0.120	0.99(0.97,1.00)	0.126
Tertiles of PNR						
T1	Reference		Reference		Reference	
T2	0.56(0.39,0.80)	0.002	0.62(0.42,0.91)	0.016	0.49(0.31,0.78)	0.003
T3	0.53(0.36,0.76)	0.001	0.73(0.48,1.09)	0.123	0.69(0.42,1.13)	0.143
HRR (continuous)	0.82(0.75,0.89)	<0.001	0.84(0.76,0.92)	<0.001	0.92(0.82,1.03)	0.147
Tertiles of HRR						
T1	Reference		Reference		Reference	
T2	0.72(0.51,1.03)	0.075	0.79(0.54,1.16)	0.223	0.87(0.56,1.37)	0.550
T3	0.34(0.23,0.50)	<0.001	0.37(0.24,0.58)	<0.001	0.62(0.36,1.05)	0.073
ALI (continuous)	0.97(0.97,0.98)	<0.001	0.98(0.97,0.99)	<0.001	0.99(0.98,1.00)	0.001*
Tertiles of ALI						
T1	Reference		Reference		Reference	
T2	0.42(0.29,0.60)	<0.001	0.52(0.35,0.76)	0.001	0.61(0.39,0.95)	0.028
T3	0.24(0.16,0.35)	<0.001	0.33(0.22,0.50)	<0.001	0.49(0.30,0.80)	0.004

Model 1, crude model; Model 2: adjusted for age≥60 and sex; Model 3: adjusted for age≥60, sex, low education, exercise, BMI≥25, smoking, drinking, duration of diabetes, HT, DR, DN, DPN, CVD, LDL-C, HDL-C, osteoporosis and SGLT-2i/GLP-1RA.

Participants were categorized into tertiles (T1, T2, T3) based on the distribution of each inflammatory indicator, with T1 representing the lowest and T3 the highest values.

*P*-values were adjusted for multiple comparisons using Bonferroni correction (20 comparisons; significance threshold, *P* < 0.0025). *P < 0.0025 (significant after Bonferroni correction).

BMI, body mass index; HT, hypertension; DR, diabetic retinopathy; DN, diabetic nephropathy; DPN, diabetic peripheral neuropathy; CVD, cardiovascular disease; LDL-C, low-density lipoprotein cholesterol; HDL-C, high-density lipoprotein cholesterol; SGLT-2i/GLP-1RA, use of either sodium-glucose-linked transporter- 2 inhibitors or glucagon-like peptide-1 receptor agonists; SII, systemic immune-inflammation index; SIRI, systemic inflammation response index; NLR, neutrophil-to-lymphocyte ratio; PIV, pan-immune inflammation value; PLR, platelet-to-lymphocyte ratio; NPAR, neutrophil-to-albumin percentage ratio; NLPR, neutrophil-lymphocyte-platelet ratio; PNR, platelet-to-neutrophil ratio; HRR, hemoglobin-to-red cell distribution width ratio; ALI, advanced lung cancer inflammation index; T2DM, type 2 diabetes mellitus;

### 3.4 Association of inflammatory indicators with severe sarcopenia by logistic regression analysis

The univariable analysis showed that SII, SIRI, PIV, NPAR, NLPR and NLR were positively associated with severe sarcopenia (*P* < 0.01), while ALI, PNR and HRR were negatively associated with severe sarcopenia (*P* < 0.05). Compared to the T1 group of SII, SIRI, PIV, NPAR, NLPR and NLR, patients in the T3 group had a significantly higher odds of severe sarcopenia (*P* < 0.01). However, compared to the T1 group of HRR, patients in the T3 group showed a significantly lower odds of severe sarcopenia (*P* < 0.01). Similarly, compared to the T1 groups of ALI and PNR, both the T2 and T3 groups exhibited significantly lower odds of severe sarcopenia (*P* < 0.01).

After full adjustment, the findings revealed that SII, SIRI, PIV, NPAR, NLPR, and NLR were positively associated with severe sarcopenia (*P* < 0.05). In contrast, ALI and PNR were inversely associated with severe sarcopenia (*P* < 0.05). After Bonferroni correction (threshold: *P* < 0.0025), ALI remained significantly associated with severe sarcopenia. Compared to the T1 group, patients in the T3 group showed significant correlations with severe sarcopenia in relation to SII, SIRI, PIV, NLR, NPAR, NLPR, and HRR after full adjustment (*P* < 0.05). Especially, compared to the T1 groups of ALI and PNR, both the T2 and T3 groups exhibited significant correlations with severe sarcopenia (*P* < 0.05) (**Table 3**).

**Table 3:** Association between inflammatory indicators and severe sarcopenia in T2DM patients.

Variables	Model 1		Model 2		Model 3	
	OR (95%CI)	*P*-value	OR (95%CI)	*P*-value	OR (95%CI)	*P*-value
SII (continuous)	1.01(1.00,1.02)	0.006	1.01(1.00,1.02)	0.002	1.01(1.00,1.02)	0.003
Tertiles of SII						
T1	Reference		Reference		Reference	
T2	1.15(0.59,2.26)	0.687	1.24(0.62,2.48)	0.545	1.17(0.58,2.39)	0.668
T3	2.90(1.61,5.41)	0.001	2.73(1.49,5.21)	0.002	2.62(1.39,5.10)	0.004
SIRI (continuous)	1.66(1.34,2.08)	<0.001	1.56(1.23,2.01)	<0.001	1.49(1.16,1.95)	0.002*
Tertiles of SIRI						
T1	Reference		Reference		Reference	
T2	1.37(0.71,2.71)	0.351	1.21(0.61,2.43)	0.594	1.28(0.63,2.65)	0.493
T3	3.09(1.70,5.88)	<0.001	2.37(1.26,4.61)	0.009	2.51(1.29,5.05)	0.008
NLR (continuous)	1.32(1.15,1.52)	<0.001	1.27(1.11,1.46)	<0.001	1.26(1.09,1.46)	0.002*
Tertiles of NLR						
T1	Reference		Reference		Reference	
T2	1.08(0.54,2.15)	0.830	0.97(0.48,1.97)	0.935	1.05(0.51,2.16)	0.897
T3	3.20(1.79,5.97)	<0.001	2.43(1.32,4.63)	0.005	2.37(1.25,4.64)	0.010
PIV (continuous)	1.01(1.00,1.02)	<0.001	1.01(1.00,1.02)	<0.001	1.01(1.00,1.02)	0.003
Tertiles of PIV						
T1	Reference		Reference		Reference	
T2	2.15(1.12,4.30)	0.024	2.25(1.15,4.58)	0.020	2.01(1.01,4.17)	0.053
T3	3.05(1.62,6.03)	0.001	2.91(1.52,5.86)	0.002	2.88(1.45,5.95)	0.003
PLR (continuous)	1.00(0.99,1.01)	0.197	1.00(1.00,1.01)	0.156	1.00(1.00,1.01)	0.375
Tertiles of PLR						
T1	Reference		Reference		Reference	
T2	1.15(0.63,2.11)	0.656	1.18(0.63,2.22)	0.602	1.17(0.61,2.24)	0.640
T3	1.62(0.91,2.92)	0.105	1.57(0.86,2.89)	0.144	1.35(0.73,2.55)	0.343
NPAR (continuous)	1.22(1.13,1.32)	<0.001	1.17(1.08,1.26)	<0.001	1.15(1.06,1.25)	0.001*
Tertiles of NPAR						
T1	Reference		Reference		Reference	
T2	1.99(1.01,4.10)	0.053	1.56(0.77,3.26)	0.225	1.51(0.74,3.21)	0.270
T3	4.24(2.25,8.47)	<0.001	2.88(1.48,5.89)	0.002	2.59(1.31,5.40)	0.008
NLPR (continuous)	1.65(1.27,2.15)	<0.001	1.42(1.08,1.89)	0.014	1.38(1.04,1.85)	0.029
Tertiles of NLPR						
T1	Reference		Reference		Reference	
T2	1.03(0.51,2.06)	0.943	0.86(0.42,1.75)	0.667	0.86(0.41,1.79)	0.684
T3	3.37(1.89,6.28)	<0.001	2.20(1.18,4.22)	0.015	2.27(1.19,4.46)	0.014
PNR (continuous)	0.98(0.97,0.99)	0.002	0.99(0.97,1.00)	0.037	0.99(0.97,1.00)	0.022
Tertiles of PNR						
T1	Reference		Reference		Reference	
T2	0.44(0.25,0.75)	0.003	0.47(0.26,0.83)	0.010	0.40(0.22,0.73)	0.003
T3	0.29(0.15,0.53)	<0.001	0.38(0.19,0.71)	0.003	0.33(0.16,0.64)	0.001*
HRR (continuous)	0.86(0.76,0.97)	0.017	0.87(0.76,1.00)	0.046	0.89(0.76,1.03)	0.115
Tertiles of HRR						
T1	Reference		Reference		Reference	
T2	1.04(0.61,1.77)	0.899	1.07(0.61,1.88)	0.816	1.01(0.56,1.80)	0.986
T3	0.37(0.19,0.71)	0.003	0.37(0.18,0.76)	0.008	0.42(0.20,0.89)	0.025
ALI (continuous)	0.97(0.96,0.98)	<0.001	0.97(0.96,0.99)	<0.001	0.98(0.97,0.99)	0.002*
Tertiles of ALI						
T1	Reference		Reference		Reference	
T2	0.33(0.19,0.58)	<0.001	0.40(0.22,0.70)	0.002	0.46(0.25,0.83)	0.010
T3	0.20(0.10,0.38)	<0.001	0.28(0.14,0.54)	<0.001	0.39(0.19,0.76)	0.008

Model 1, crude model; Model 2, adjusted for age≥60 and sex; Model 3, adjusted for age≥60, sex, low education, BMI≥25, duration of diabetes, CVD and osteoporosis.

Participants were categorized into tertiles (T1, T2, T3) based on the distribution of each inflammatory indicator, with T1 representing the lowest and T3 the highest values.

*P*-values were adjusted for multiple comparisons using Bonferroni correction (20 comparisons; significance threshold, *P* < 0.0025). *P < 0.0025 (significant after Bonferroni correction).

BMI, body mass index; CVD, cardiovascular disease; SII, systemic immune-inflammation index; SIRI, systemic inflammation response index; NLR, neutrophil-to-lymphocyte ratio; PIV, pan-immune inflammation value; PLR, platelet-to-lymphocyte ratio; NPAR, neutrophil-to- albumin percentage ratio; NLPR, neutrophil-lymphocyte-platelet ratio; PNR, platelet-to-neutrophil ratio; HRR, hemoglobin-to-red cell distribution width ratio; ALI, advanced lung cancer inflammation index; T2DM, type 2 diabetes mellitus.

### 3.5 Diagnostic performance of composite inflammatory indicators

ROC curve analysis was used to assess the utility of composite inflammatory indicators in identifying sarcopenia and severe sarcopenia among T2DM patients (**Table 4**). For sarcopenia, an ALI cutoff value of 50.29 yielded a sensitivity of 65.9% and a specificity of 61.4%, with an AUC of 0.68 (95% CI: 0.64–0.72) and a Youden’s index of 0.273. For severe sarcopenia, an ALI cutoff of 38.52 achieved a sensitivity of 56.4% and a specificity of 65.1%, with an AUC of 0.70 (95% CI: 0.63-0.76) and a Youden’s index of 0.333. To assess the robustness of the optimal cutoffs, we performed bootstrap internal validation with 1,000 resamples. The bootstrap-derived 95% confidence intervals for the sarcopenia cutoff (49.46) and severe sarcopenia cutoff (39.89) were 47.79-51.13 and 33.72-46.06, respectively, indicating good stability of our estimates. **Table 5** presents the corresponding positive/negative predictive values and likelihood ratios for both cutoffs. DeLong’s test further demonstrated that the ALI had a significantly higher AUC than mostly other inflammatory markers (all *P* < 0.05). Furthermore, after Bonferroni correction for 18 comparisons, the differences mostly remained statistically significant (*P* < 0.0028) (**Table 6**).

**Table 4:** Diagnostic performance of composite inflammatory indicators in identifying sarcopenia and severe sarcopenia among T2DM patients.

Variables	Best thresholds	Sensitivity	Specificity	AUC(95%CI)	Youden’s index
Sarcopenia					
SII	576.20	0.433	0.745	0.60(0.55-0.64)	0.178
SIRI	1.33	0.341	0.848	0.61(0.57-0.65)	0.189
NLR	2.68	0.387	0.814	0.61(0.57-0.65)	0.201
PIV	286.32	0.356	0.804	0.60(0.56-0.64)	0.160
PLR	112.32	0.598	0.550	0.58(0.54-0.62)	0.148
NPAR	16.96	0.379	0.850	0.65(0.61-0.69)	0.229
NLPR	1.11	0.479	0.715	0.61(0.57-0.66)	0.194
PNR	48.31	0.456	0.701	0.59(0.54-0.63)	0.273
HRR	11.76	0.770	0.432	0.62(0.58-0.66)	0.157
ALI	50.29	0.659	0.614	0.68(0.64-0.72)	0.273
Severe sarcopenia					
SII	567.80	0.513	0.722	0.62(0.55-0.69)	0.235
SIRI	1.33	0.423	0.840	0.64(0.57-0.71)	0.263
NLR	2.82	0.436	0.835	0.64(0.57-0.71)	0.271
PIV	143.93	0.821	0.409	0.63(0.56-0.70)	0.230
PLR	125.37	0.462	0.659	0.56(0.49-0.63)	0.121
NPAR	15.35	0.654	0.651	0.68(0.62-0.75)	0.305
NLPR	1.16	0.551	0.742	0.65(0.58-0.72)	0.293
PNR	55.83	0.718	0.547	0.64(0.58-0.71)	0.265
HRR	11.80	0.821	0.406	0.60(0.54-0.67)	0.227
ALI	38.52	0.564	0.769	0.70(0.63-0.76)	0.333

SII, systemic immune-inflammation index; SIRI, systemic inflammation response index; NLR, neutrophil-to-lymphocyte ratio; PIV, pan-immune inflammation value; PLR, platelet-to-lymphocyte ratio; NPAR, neutrophil-to-albumin percentage ratio; NLPR, neutrophil-lymphocyte-platelet ratio; PNR, platelet-to-neutrophil ratio; HRR, hemoglobin-to-red cell distribution width ratio; ALI, advanced lung cancer inflammation index; T2DM, type 2 diabetes mellitus.

**Table 5:** The diagnostic performance of the ALI cutoffs in identifying sarcopenia and severe sarcopenia among T2DM patients.

Variables	Sarcopenia	Severe sarcopenia
Sensitivity	0.659	0.564
Specificity	0.614	0.769
AUC(95%CI)	0.68 (0.64-0.72)	0.70 (0.63-0.76)
Cutoff	50.29	38.52
Bootstrap mean cutoff (95%CI)	49.46 (47.79-52.13)	39.89 (33.72-47.06)
PPV	47.9%	24.3%
NPV	77.9%	92.7%
+LR	1.71	2.44
-LR	0.56	0.57

ALI, advanced lung cancer inflammation index; T2DM, type 2 diabetes mellitus; PPV, positive predictive value; NPV, negative predictive value; +LR, positive likelihood ratio; -LR, negative likelihood ratio.

**Table 6:** DeLong’s test for pairwise AUC comparisons between ALI and other inflammatory indicators for sarcopenia and severe sarcopenia.

Variables	AUC difference (95%CI)	*P*-value
Sarcopenia		
ALI vs SII	0.08 (0.06-0.11)	<0.001*
ALI vs SIRI	0.07 (0.05-0.10)	<0.001*
ALI vs NLR	0.07 (0.06-0.09)	<0.001*
ALI vs PIV	0.08 (0.05-0.12)	<0.001*
ALI vs PLR	0.10 (0.07-0.14)	<0.001*
ALI vs NPAR	0.03 (0.02-0.06)	<0.001*
ALI vs NLPR	0.07 (0.05-0.10)	<0.001*
ALI vs PNR	0.09 (0.06-0.14)	<0.001*
ALI vs HRR	0.06 (0.01-0.12)	0.015
Severe sarcopenia		
ALI vs SII	0.08 (0.04-0.12)	<0.001*
ALI vs SIRI	0.06 (0.02-0.09)	0.002*
ALI vs NLR	0.06 (0.03-0.08)	<0.001*
ALI vs PIV	0.07 (0.03-0.11)	<0.001*
ALI vs PLR	0.14 (0.07-0.20)	<0.001*
ALI vs NPAR	0.02 (-0.02-0.04)	0.415
ALI vs NLPR	0.05 (0.01-0.09)	0.042
ALI vs PNR	0.06 (-0.01-0.11)	0.062
ALI vs HRR	0.10 (0.01-0.18)	0.028

*P values were adjusted for multiple comparisons using Bonferroni correction (18 tests in total; significance threshold, *P* < 0.0028).

SII, systemic immune-inflammation index; SIRI, systemic inflammation response index; NLR, neutrophil-to-lymphocyte ratio; PIV, pan-immune inflammation value; PLR, platelet-to-lymphocyte ratio; NPAR, neutrophil-to-albumin percentage ratio; NLPR, neutrophil-lymphocyte-platelet ratio; PNR, platelet-to-neutrophil ratio; HRR, hemoglobin-to-red cell distribution width ratio; ALI, advanced lung cancer inflammation index.

### 3.6 Decision curve analysis

**Figure 4:**
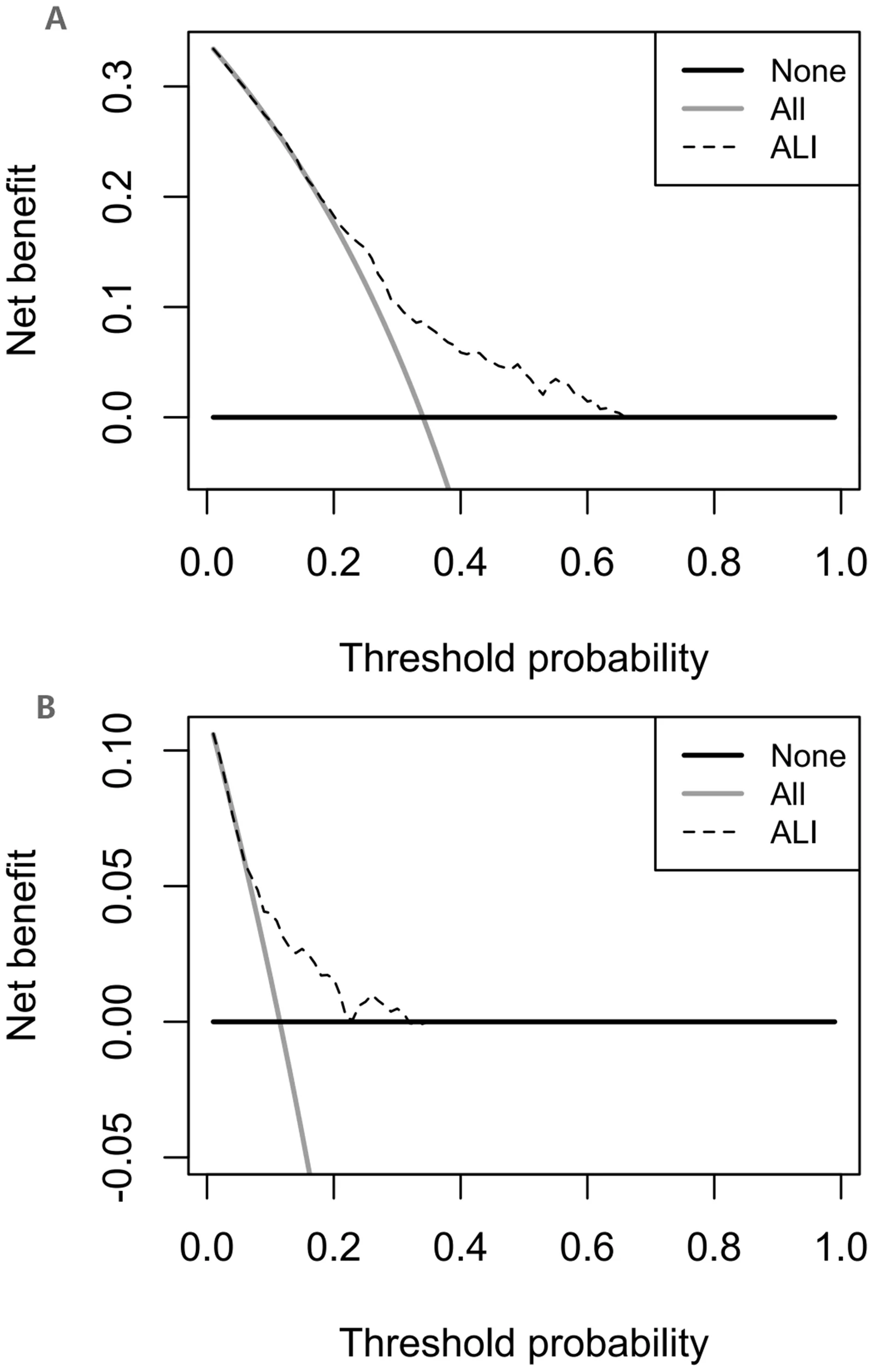
Decision curve analysis for ALI in predicting sarcopenia and severe sarcopenia. **(A)** Decision curve analysis for ALI in predicting sarcopenia. **(B)** Decision curve analysis for ALI in predicting severe sarcopenia.

The DCA demonstrated that the ALI provided a positive net benefit for predicting both sarcopenia and severe sarcopenia (**Figure 4**). For sarcopenia, the ALI model showed a higher net benefit than the “treat-all” and “treat-none” strategies across a threshold range of approximately 0.2-0.7. For severe sarcopenia, the model maintained a net benefit advantage over the two reference strategies within a narrower range of approximately 0.1-0.4, beyond which the net benefit converged toward the “treat-none” strategy.

## 4 Discussion

This is the first study to analyze the relationship between composite inflammatory indicators and both sarcopenia and severe sarcopenia in patients with T2DM. This study demonstrated significant positive correlations between elevated levels of composite inflammatory indicators (SII, SIRI, PIV, NPAR, NLPR, and NLR) and sarcopenia in patients with T2DM, whereas ALI was inversely associated with sarcopenia. These associations remained significant in T2DM patients with severe sarcopenia. ALI outperformed other composite inflammatory indicators in identifying sarcopenia and severe sarcopenia in patients with T2DM.

The association between composite inflammatory indicators and sarcopenia was investigated in previous studies. In the general population, Gou et al. found SII, SIRI, and NLR to be independent predictors of sarcopenia (9). In a cross-sectional study of 272 patients undergoing maintenance hemodialysis, NLR was significantly associated with sarcopenia risk among overweight individuals, even after adjustment for potential covariates (44). Long et al. reported that high PIV levels are an independent risk factor for sarcopenia in hypertensive patients, with the prevalence of sarcopenia showing a positive correlation with PIV levels (10). Meanwhile, a prospective multicenter study demonstrated that a decline in ALI has significant predictive value for sarcopenia occurrence in cancer patients (14). In line with previous studies, Our study revealed that elevated levels of SII, SIRI, NLR, and PIV were positively associated with sarcopenia, whereas ALI was negatively associated with sarcopenia in T2DM patients. This consistency suggests that systemic inflammation, as reflected by these composite indices, may contribute to the development of sarcopenia across various chronic disease states, including type 2 diabetes. In addition to the composite inflammation indicators mentioned above, NPAR and NLPR have also demonstrated predictive utility in various chronic inflammatory conditions. Elevated NPAR has been robustly associated with incident diabetic retinopathy and nephropathy progression (21, 22). Zhao et al. revealed a dose-dependent relationship between elevated NLPR levels and increased all-cause mortality in stroke cohorts (45). In this study, we found that elevated NPAR and NLPR levels correlated positively with sarcopenia. These results showed that chronic inflammation, as measured by NPAR and NLPR, was linked to sarcopenia in T2DM patients. This finding extended the previously established relationship between composite inflammation indicators and chronic inflammatory diseases to sarcopenia.

A previous study showed that PLR was associated with sarcopenia risk in post-nephrectomy patients (15). Another study demonstrated that elevated PLR levels were associated with a higher risk of sarcopenia in geriatric populations (16). HRR, as an indicator of inflammation and oxidative stress, can serve as a simple yet effective biomarker for predicting outcomes and assessing patient status in various medical conditions. A study from the nationally representative National Health and Nutrition Examination Survey (NHANES) (2011–2018) showed that lower HRR levels may be an independent risk factor for sarcopenia (31). However, our study found that when PLR was analyzed as continuous or categorical variable, patients in the T2 group showed no significant correlation with sarcopenia compared to the T1 group, whereas a significant correlation was observed for the T3 group. When HRR was analyzed as either continuous or categorical variable, no significant association with sarcopenia was observed. These discrepancies likely arise from differences in study populations, sample sizes, and sarcopenia diagnostic criteria. Larger prospective cohorts are needed to clarify the relationship between PLR, HRR and sarcopenia in T2DM patients.

Severe sarcopenia is associated with significantly higher risks of all-cause mortality, cardiovascular disease, and respiratory disease (46). However, systematic investigations regarding the association between inflammatory indicators and severe sarcopenia remain scarce. Our study extended the link between inflammatory indicators and sarcopenia to the severe form of the condition. Our results revealed that elevated levels of SII, SIRI, NLR, PIV, NPAR, and NLPR were significantly positively correlated with severe sarcopenia, whereas ALI exhibited an inverse association. This is consistent with the findings of sarcopenia. Notably, we further found that when analyzed as a continuous or categorical variable, PNR showed no significant correlation with sarcopenia in T2DM patients. However, PNR exhibited a significant negative correlation with severe sarcopenia, suggesting that a lower PNR may reflect a state of neutrophilia-dominated inflammation that is particularly detrimental to muscle homeostasis. In patients with T2DM, aberrant levels of inflammatory indicators may signal the concurrent presence of severe sarcopenia. Therefore, comprehensive identifying for severe sarcopenia should be performed in these high-risk patients according to the AWGS guidelines, including muscle mass, grip strength, and walking speed measurements.

Among the ten composite inflammatory indicators evaluated, compared to T1, only ALI in both T2 and T3 groups exhibited significant association with sarcopenia and severe sarcopenia in T2DM patients. The results of the ROC curve analysis indicated that ALI was the most predictive parameter. Therefore, we consider ALI superior to other indicators in identifying sarcopenia and severe sarcopenia in T2DM patients. This advantage may stem from ALI’s unique composition, which integrates adiposity, nutritional status, and systemic inflammation into a single multimodal biomarker (BMI × albumin/NLR). Clinically, reduced ALI values may indicate a catabolic state characterized by malnutrition, chronic low-grade inflammation, and impaired muscle protein synthesis. Obesity promotes immune cell infiltration into skeletal muscle and induces a pro-inflammatory state in both myocytes and perimuscular adipose tissue, thereby exacerbating myocyte inflammation. Additionally, it drives intramyocellular ectopic lipid accumulation, increases mitochondrial oxidative stress, and leads to subsequent metabolic disturbances (47–49). As a standard nutritional marker, albumin demonstrates clinical relevance, as evidenced by its inverse association with diabetes incidence and complications (50, 51). This relationship is further supported by its inherent anti-inflammatory properties, reflected in the observed decrease of pro-inflammatory cytokines (e.g., TNF, CRP) associated with higher albumin levels (47, 52). Hypoalbuminemia during inflammatory states may exacerbate muscle wasting by suppressing protein synthesis (47, 52). NLR reflects a pro-inflammatory microenvironment (neutrophil-derived IL-6, TNF-α) coupled with lymphopenia-induced immunosuppression, thereby accelerating proteolysis (40). Although ALI demonstrated the best discriminative ability among the ten inflammatory indicators evaluated, its AUC of 0.68-0.70 indicated only modest predictive power for identifying sarcopenia and severe sarcopenia in T2DM patients. This suggests that while ALI may serve as a useful identifying tool in clinical settings, its diagnostic utility alone is limited. However, DCA demonstrated that the ALI cutoff of 50.29 provided a positive net clinical benefit across a range of threshold probabilities (approximately 0.2-0.7), indicating that using ALI to guide clinical decision-making for sarcopenia identifying would improve patient outcomes. Future studies should therefore explore biomarker combinations or integration with other clinical parameters to improve predictive accuracy.

Our study suggests that early identifying for sarcopenia should be initiated in hospitalized T2DM patients with low ALI levels. Given that ALI’s components are routinely available in standard clinical practice, it offers a cost-effective and practical tool. Consequently, ALI may serve as a useful indicator for sarcopenia in diabetic clinics. Patients identified with low ALI values could be prioritized for more comprehensive sarcopenia assessments, including DXA for muscle mass and tests for muscle strength and physical performance, as recommended by the AWGS guidelines. For high-risk populations, potential interventions include nutritional support (e.g., supplements rich in curcumin and leucine metabolites), resistance training, and other complementary approaches (53–55). Future studies should further investigate the application of ALI in outpatient settings.

The strength of this study lies in its comprehensive examination of the associations between composite inflammatory indicators and both sarcopenia and severe sarcopenia in patients with T2DM. Nevertheless, several limitations warrant consideration. First, the cross-sectional nature of this study prevents causal inference regarding the relationship between inflammatory markers and sarcopenia. Longitudinal or interventional studies are therefore required to determine temporal causality. Second, the single-center inpatient design in China, coupled with the poor glycemic status of our populations (mean HbA1c 9.8%), limits the generalizability of our findings to broader community/outpatient populations and to other ethnic or geographic settings. Third, no comprehensive data were available regarding dietary habits, which are important determinants of muscle mass. Additionally, we did not assess gonadal function, a factor relevant to musculoskeletal health, and this omission precluded exploration of its potential interaction with inflammation in sarcopenia pathogenesis.

## 5 Conclusion

Our study identified that SII, SIRI, PIV, NPAR, NLPR, and NLR were linked to elevated odds of sarcopenia in T2DM, whereas ALI exhibited a negative correlation. Notably, ALI outperformed the other nine indicators in our study. Low ALI in T2DM patients should trigger prioritized identifying for sarcopenia in clinical settings. Prospective studies are warranted to investigate whether low ALI is prospectively associated with sarcopenia development, and whether anti-inflammatory interventions may mitigate the incidence and progression of sarcopenia in T2DM patients.

## Data Availability

The original contributions presented in the study are included in the article/supplementary material. Further inquiries can be directed to the corresponding authors.
